# Supplementary material for: Association vs. Prediction: The Impact of Cortical Surface Smoothing and Parcellation on Brain Age
Source: Front Big Data. 2021 May 4;4:637724. doi: 10.3389/fdata.2021.637724 (PMC8131952; doi:10.3389/fdata.2021.637724)
Supplement: Supplementary file 1 [file Data_Sheet_1.PDF]

Supplementary Material

Supplementary Method – Estimating the number of resels:

Number of resolution elements (i.e. resel numbers) is defined to identify the independent elements of the statistical results. The resel numbers and roughness/smoothness of a map are dependent. In theory, resel numbers are defined as determinants of the variance-covariance matrix of the gradient of the component fields based on the statistical results (Worsley et al., 1992). However, in practice, Worsley et al. proposed a simplified estimation which is calculated across the edges across the surface for each of the statistical maps and provides a robust estimation of the resels (implemented in the SurfStat software https://www.math.mcgill.ca/keith/surfstat/). We have used this implementation to estimate resel numbers across smoothing and parcellation conditions for each statistical map.

1 Supplementary Figures

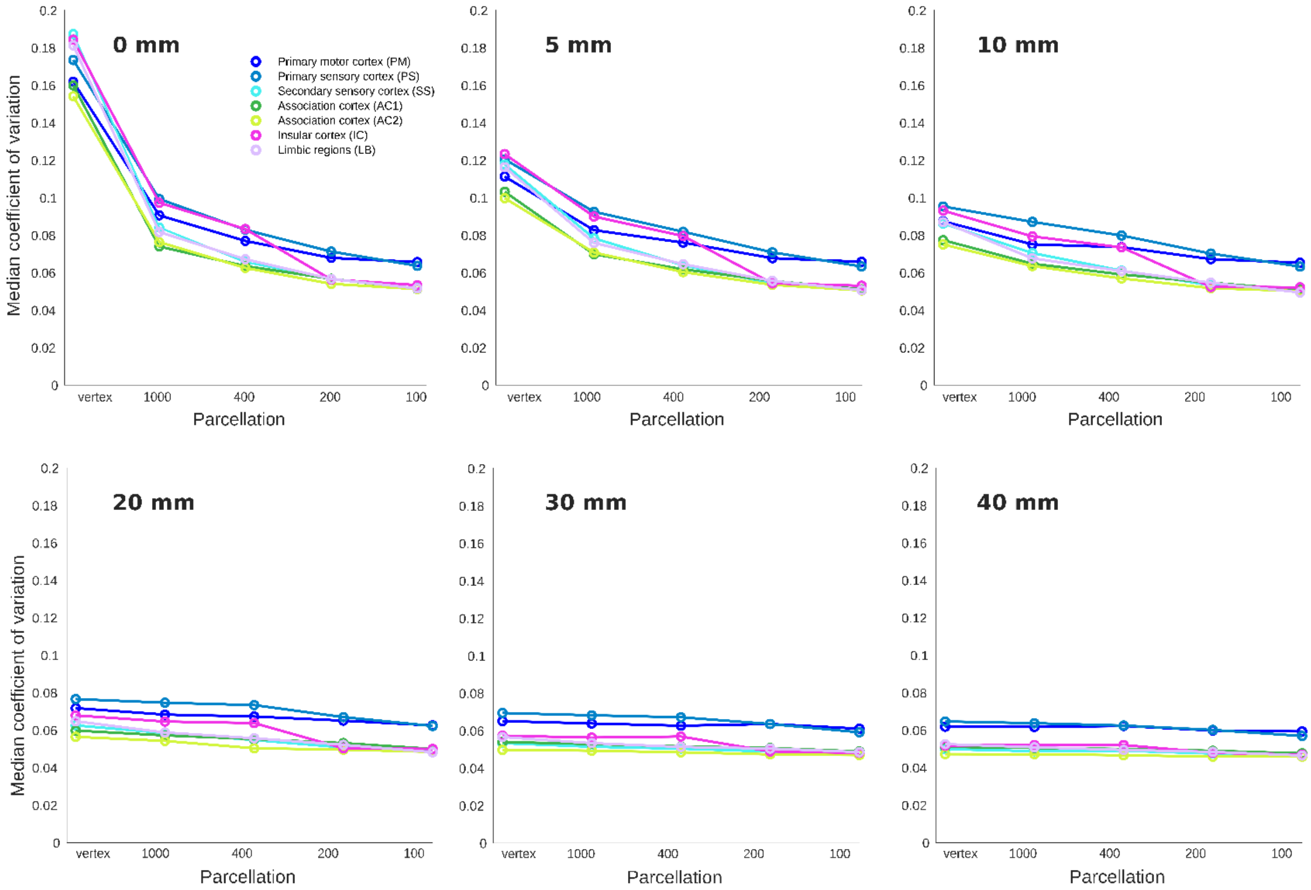

Supplementary Figure 1. Coefficient of variation (CV) shown for each cytoarchitectural region across parcellation and smoothing conditions.

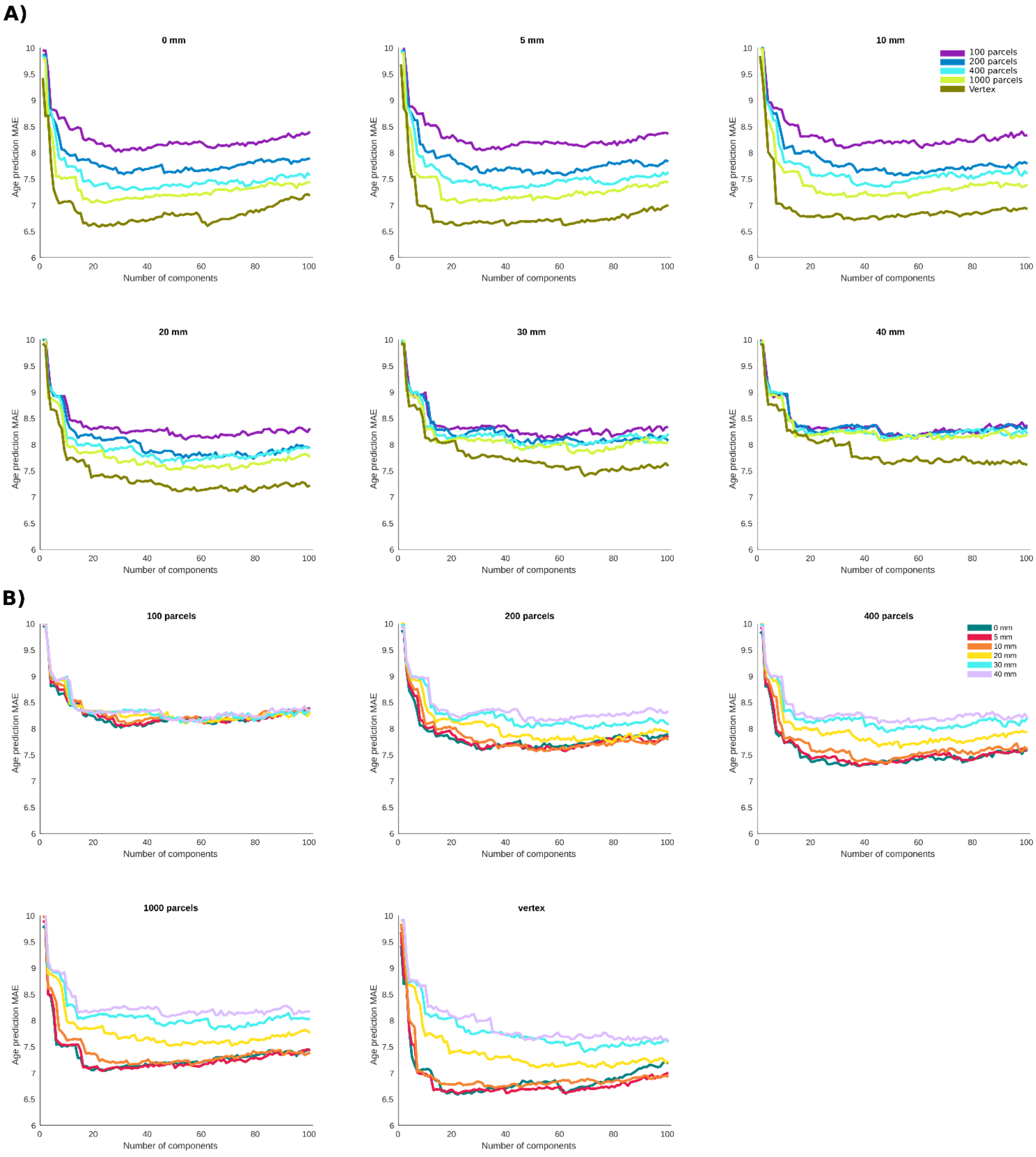

Supplementary Figure 2. Mean absolute error (MAE) for linear regression model. Age prediction as a function of number of principal components included as features in the predictive model. **A)** results grouped together based on the smoothing level. **B)** results grouped together based on the parcellation resolution.

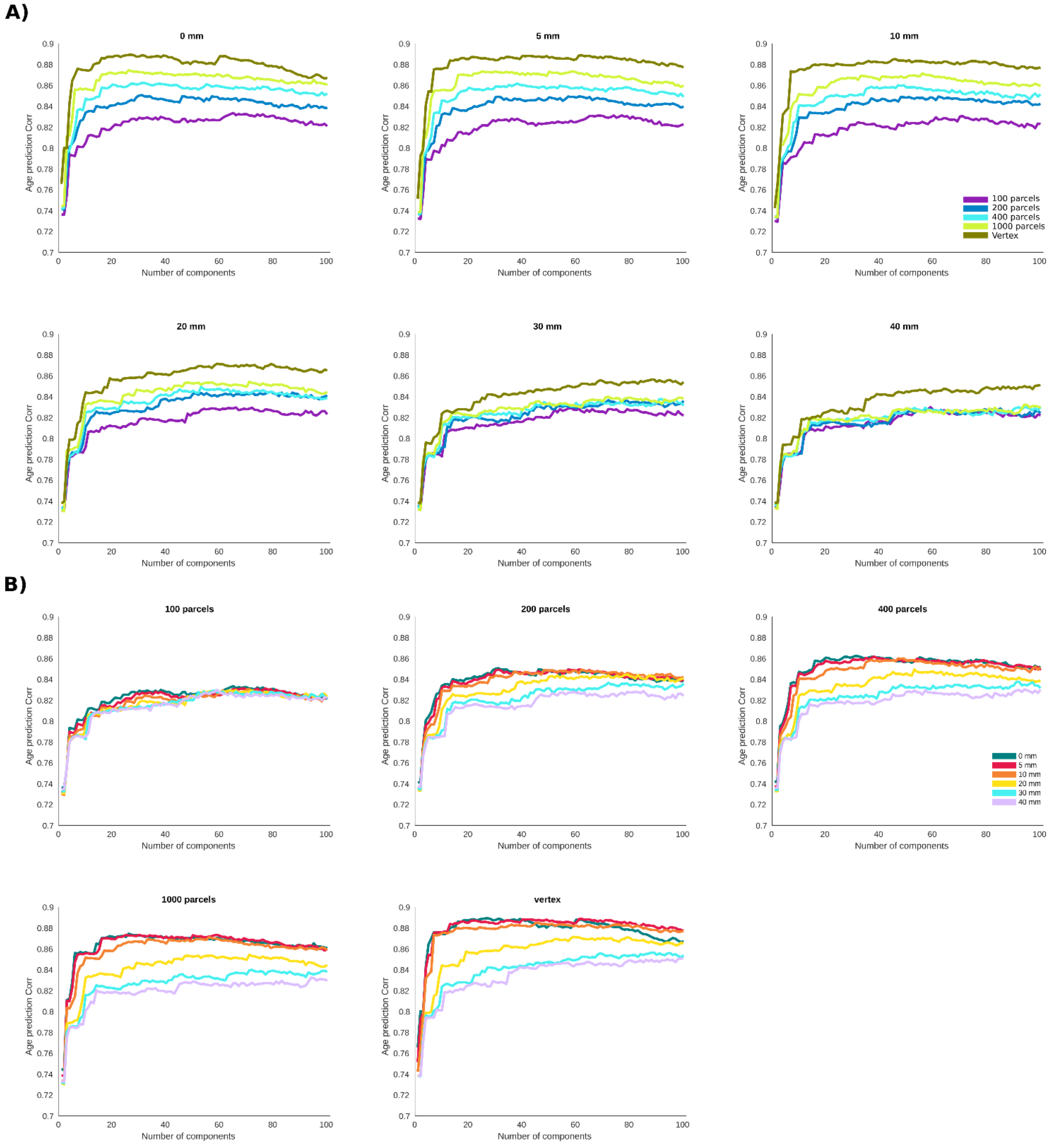

Supplementary Figure 3. Correlation between the predicted age and chronological age as a function of number of principal components included as features in the predictive model. **A)** results grouped together based on the smoothing level. **B)** results grouped together based on the parcellation resolution.

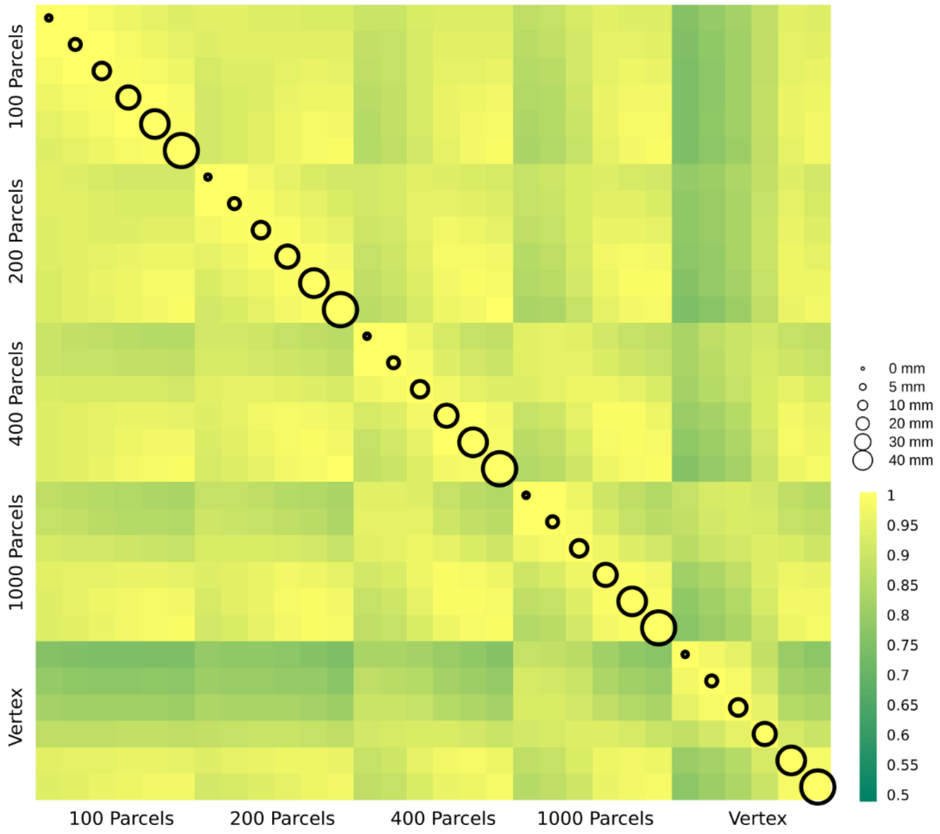

Supplementary Figure 4. δ_1 age prediction error. The correlation between delta age (as measured by δ_1) across parcellation resolutions (x axis labels) and smoothing kernels (represented by circle size).

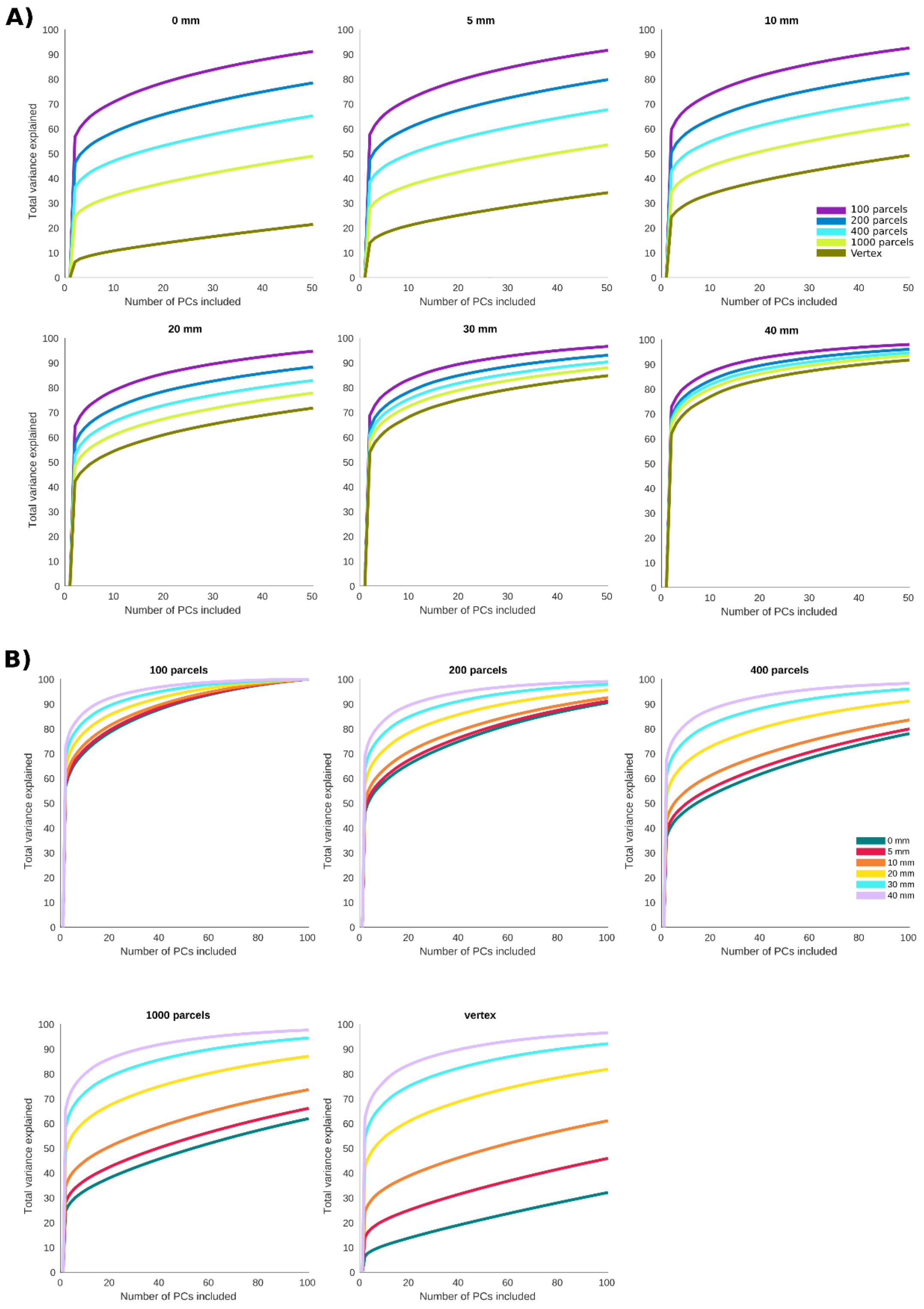

Supplementary Figure 5. Accumulated variance explained of cortical thickness by principal components for each smoothing/parcellation pair. **A)** results grouped together based on the smoothing level. **B)** results grouped together based on the parcellation resolution.

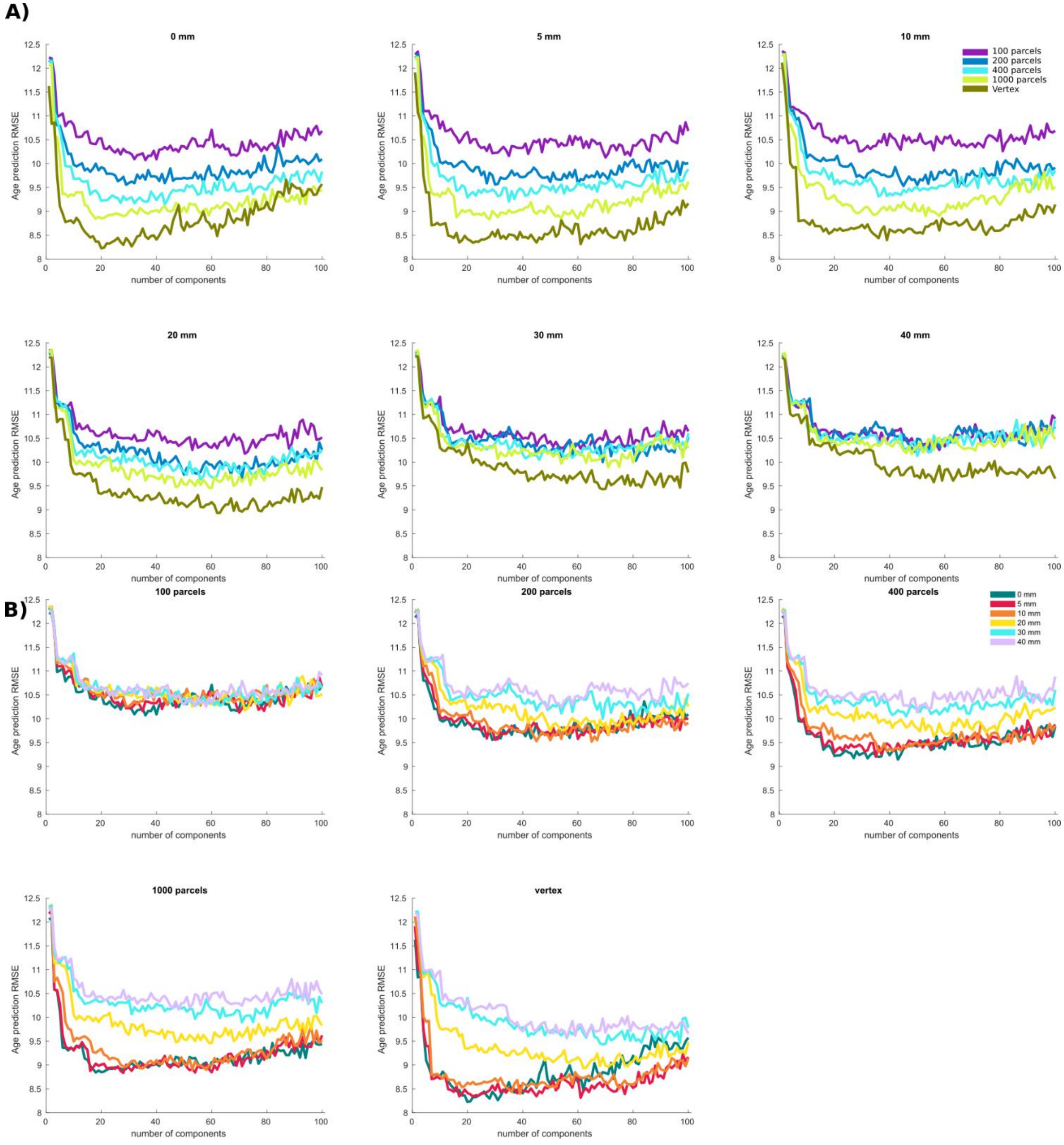

Supplementary Figure 6. Root mean square error (RMSE) for Age prediction using support vector machine regression model with linear kernel as a function of number of principal components included as features in the predictive model. **A)** results grouped together based on the smoothing level. **B)** results grouped together based on the parcellation resolution.

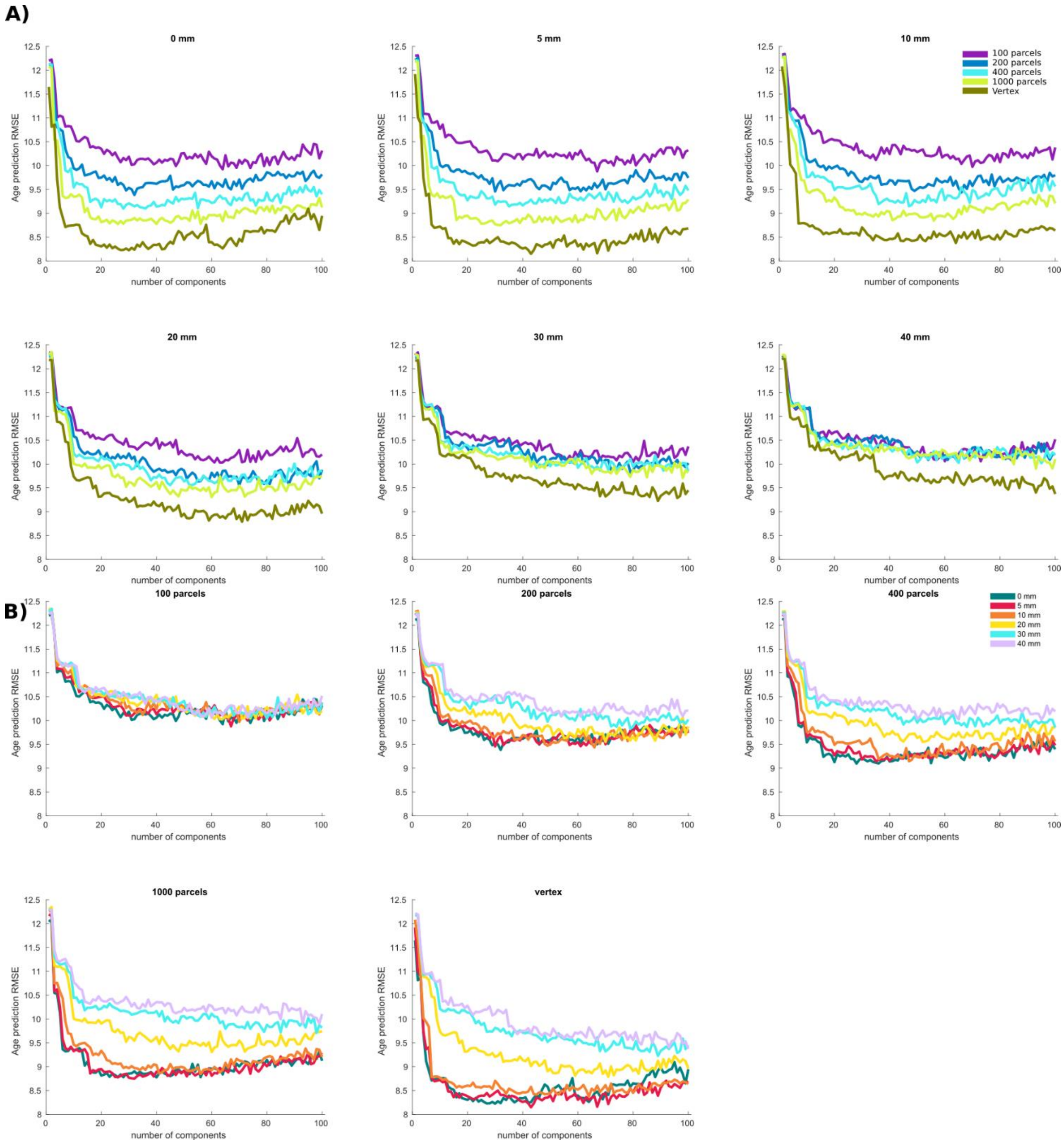

Supplementary Figure 7. Root mean square error (RMSE) for Age prediction using linear regression model with ridge regularization (λ = 0.0215) as a function of number of principal components included as features in the predictive model. **A)** results grouped together based on the smoothing level. **B)** results grouped together based on the parcellation resolution.

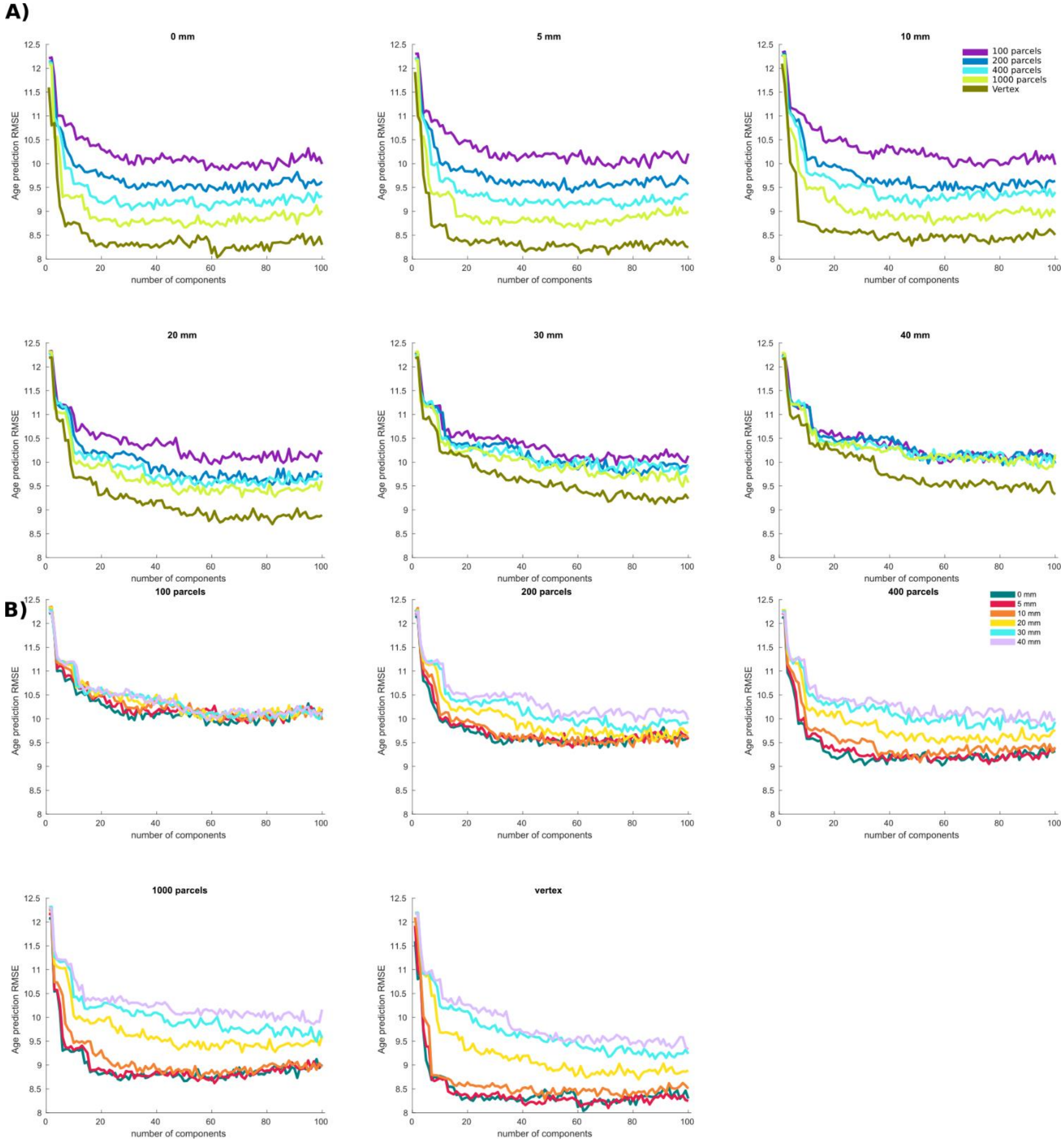

Supplementary Figure 8. Root mean square error (RMSE) for Age prediction using linear regression model with lasso regularization ($\lambda = 0.1$) as a function of number of principal components included as features in the predictive model. **A)** results grouped together based on the smoothing level. **B)** results grouped together based on the parcellation resolution.

Supplementary Table 1. Root mean square error (RMSE) for Age prediction using linear regression model and PCA-based cortical thickness features. Each column includes a smoothing-parcellation pair condition and each row includes the number of PCs used as predictors in the model.

Supplementary Table 2. Root mean square error (RMSE) for Age prediction using support vector machine regression model with linear kernel and PCA-based cortical thickness features. Each column includes a smoothing-parcellation pair condition and each row includes the number of PCs used as predictors in the model.

Supplementary Table 3. Root mean square error (RMSE) for Age prediction using linear regression model with ridge regularization (each λ in a separate sheet) using PCA-based cortical thickness features. Each column includes a smoothing-parcellation pair condition and each row includes the number of PCs used as predictors in the model.

Supplementary Table 4. Root mean square error (RMSE) for Age prediction using linear regression model with lasso regularization (each λ in a separate sheet) using PCA-based cortical thickness features. Each column includes a smoothing-parcellation pair condition and each row includes the number of PCs used as predictors in the model.

Supplementary Table 5. PCA projection for the first 100 PC components of cortical thickness mapped in the subject space used as features in the model across conditions.

Supplementary Table 6. PCA projection for the first 100 PC components of cortical thickness mapped on the Schaefer brain parcellation and CIVET surface.
