# Supplementary material for: Association vs. Prediction: The Impact of Cortical Surface Smoothing and Parcellation on Brain Age
Source: Front Big Data. 2021 May 4;4:637724. doi: 10.3389/fdata.2021.637724 (PMC8131952; doi:10.3389/fdata.2021.637724)
Supplement: Supplementary file 2 [file Data_Sheet_2.PDF]

Supplementary Table 6:

https://www.dropbox.com/scl/fi/xhjrstaj5l2ljb9izjukh/X_all_pca_coeff.xlsx?dl=0&rlkey=upoclja1i7b5m317hky4v9fpb
